# Health equity issues at the local level: Socio-geography, access, and health outcomes in the service area of the Hôpital Albert Schweitzer-Haiti

**DOI:** 10.1186/1475-9276-6-7

**Published:** 2007-08-01

**Authors:** Henry B Perry, Leslie W King-Schultz, Asma S Aftab, John H Bryant

**Affiliations:** 1 Future Generations, Franklin, WV, USA; 2 Mayo Medical School, Mayo Clinic College of Medicine, Rochester, MN, USA; 3 Department of Family Medicine and Community Health, University of Miami, Miller, School of Medicine, FL, USA; 4 Bloomberg School of Public Health, Johns Hopkins University, Baltimore, MD, USA

## Abstract

**Background:**

Although health equity issues at regional, national and international levels are receiving increasing attention, health equity issues at the local level have been virtually overlooked. Here, we describe here a comprehensive equity assessment carried out by the Hôpital Albert Schweitzer-Haiti (HAS) in 2003. HAS has been operating health and development programs in the Artibonite Valley of Haiti for 50 years.

**Methods:**

We reviewed all available information arising from a comprehensive evaluation of the programs of HAS carried out in 1999 and 2000. As part of this evaluation, two demographic and health surveys were carried out. We carried out exit interviews with clients receiving primary health care, observations within health facilities, interviews with households related to quality of care, and focus group discussions with community-based health workers. A special study was carried out in 2003 to assess factors determining the use of prenatal care services. Finally, selected findings were obtained from the HAS information system.

**Results:**

We found markedly reduced access to health services in the peripheral mountainous areas compared to the central plains. The quality of services was more deficient and the coverage of key services was lower in the mountains. Finally, health status, as measured by under-five mortality rates and levels of childhood malnutrition, was also worse in the mountains.

**Conclusion:**

These findings indicate that local health programs need to give attention to monitoring the health status as well as the quality and coverage of basic services among marginalized groups within the program service area. Health inequities will not be overcome until such monitoring occurs and leaders of health programs ensure that inequities identified are addressed in the local programming of activities. It is quite likely that, within relatively small geographic areas in resource-poor settings around the world, similar, if not even greater, levels of health inequities exist. These inequities need to be measured and addressed in order for health programs to achieve equity and maximum improvement in health status within the population.

## Background

Issues of inequities in health have received increased attention in recent years. Many discussions of health equity focus on the disparities of health program inputs such as staff and services relative to need, all of which are avoidable and therefore unjust [1-6]. To a lesser degree in the literature, inequity in health also refers to differences in health outcomes that are avoidable and therefore unjust. The underlying social determinants which lead to poorer health outcomes among the disadvantaged need to be given special attention in order to reduce inequities in health status [7,8].

In an equitable system, those individuals with the poorest health status should receive more health care and other services to address their greater need. The increased attention now being given to reducing health inequities arises from the obvious fact that, even though the opportunity to be healthy is a basic and universal human right, health systems are failing to reach the poorest of the poor, health disparities are increasing, and the opportunity for a healthy life is denied to billions of people around the world [9-11].

The challenge of addressing health inequities is especially great for developing nations whose limited resources and inadequate data collection systems create large hurdles for establishing effective interventions aimed at reducing inequities. Haiti, for instance, is the most underdeveloped nation in the Western hemisphere, with a per capita GNP of only US$415 (less than half that of the next poorest country in the Western hemisphere) and an infant mortality rate of 79 per 1000 live births (more than twice that of the other poor nations in the Western hemisphere except for Bolivia's, which is almost one-third less) [12]. Likewise, access to medical care in Haiti is limited, with only 25 physicians per 100,000 population (compared to at least four times that many in virtually all of the other poor countries of the Western hemisphere) and only 24% of births attended by skilled health personnel (compared to 41% for the country in the Western hemisphere with the next-lowest percentage).

Wide disparities in health status exist within the borders of Haiti as well. Rural areas of Haiti, and particularly mountainous zones with Haiti, have poorer quality of health (in terms of under-five mortality and levels of childhood malnutrition) and less access to care. It is estimated that 40% of persons living in rural areas of Haiti have no access to any primary health care services. Likewise, these populations have lower rates of child immunizations and lower rates of prenatal care [13].

Much attention has been directed at global [14-16] and national [17-19] inequities that currently exist – both in terms of access to health services and health status. Also, considerable attention has been given to the concept of health inequity and how to measure it [20,21]. However, much less attention has been given to health inequities at the local level. Here, we reported findings of an equity assessment at one local level, the geographic area served by the programs of the Hôpital Albert Schweitzer in Haiti.

### The Hôpital Albert Schweitzer-Haiti

**Figure 1:**
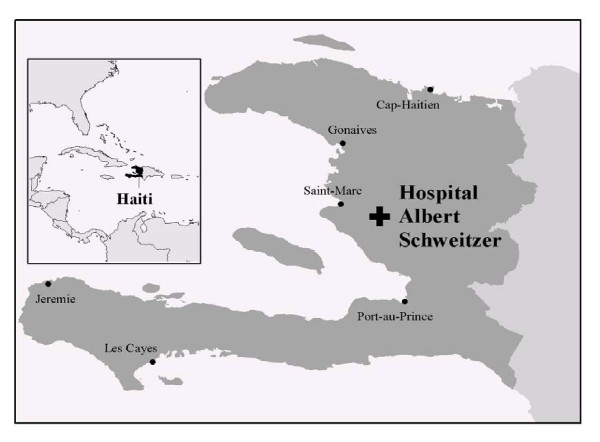
Map of the Caribbean, Haiti, and the Hôpital Albert Schweitzer Service Area.

The Hôpital Albert Schweitzer-Haiti (HAS), based in Deschapelles, Haiti (Figure 1), has been addressing the problems of equity in its service area since 1956 as part of its commitment to improve the health and quality of life of all of the people who reside in its catchment area in the rural Artibonite Valley. HAS is operated by the Grant Foundation, a not-for-profit organization registered in the United States which has a long-term contract with the Government of Haiti [21-29]. Dr. and Mrs. Larimer Mellon, following in the footsteps of their mentor Dr. Albert Schweitzer and seeking to implement Schweitzer's philosophy of "Reverence for Life", established HAS to provide compassionate and competent services to a poor rural population [30,31]. The Board of Directors declared in 1994 that the operation of programs at HAS should be directed to "improve ways to identify and select needs, assure that services are equally available to all, and assure that those with greatest need are served."

**Figure 2:**
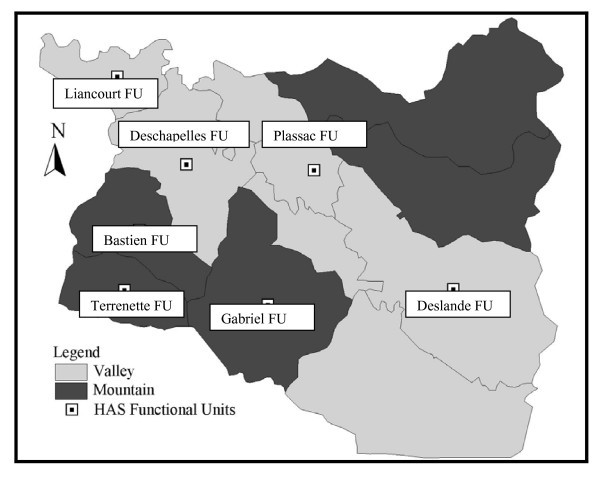
Map of the 14 Functional Units in the Health District Where the Hôpital Albert Schweitzer Operates Its Programs, the Central Valley and Mountainous Areas within the District, and the Seven Functional Units Operated by HAS.

At the time of this assessment, HAS was providing community-based primary health care services to a population of 148,000 persons (hereafter referred to as the HAS service area) in seven Functional Units of the Health District (*Unite Communale de Sante*, hereafter referred to as the UCS District) of Petite Riviere, Verettes and LaChapelle. The UCS District had at that time a total of 258,000 residents in 14 Functional Units. The HAS Hospital serves as the referral center for the entire UCS District (Figure 2). Other NGOs working with the Ministry of Health provide primary health care services in the other seven Functional Units and all health care providers in the District work closely through a UCS Coordinating Committee to create what most informed observers consider to be the most effectively functioning UCS in Haiti.

**Figure 3:**
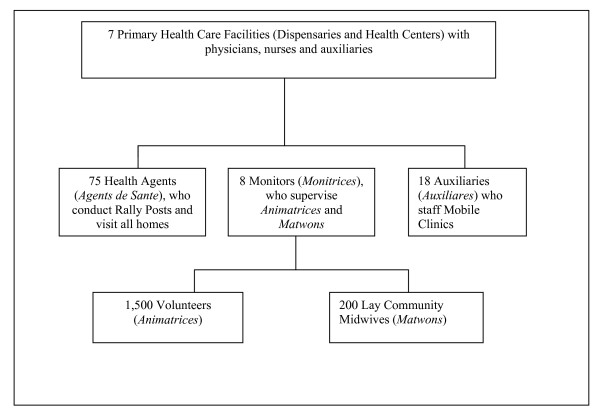
Description of the Primary Health Care Team at Hôpital Albert Schweitzer Haiti as of 2002.

The community-based primary health care program consisted of the following staff at the time of this equity assessment (see also Figure 3):

• 1,500 *Animatrices *(volunteer community health workers), one for every 15 households, who were providing peer-to-peer health education, assisting with referral to higher levels of care, and promoting community involvement in planning, implementation and evaluation of services;

• 200 *Matwons *(unsalaried community lay midwives receiving payment directly from clients) who received training in safe delivery and supervisory support from HAS;

• 75 *Agents de Sante *(Health Agents), one for approximately every 400 households, who made regular home visits and directed monthly Rally Posts for immunization, growth monitoring/nutritional counseling, and referral;

• 8 *Monitrices *(Monitors), who provided liaison with and training of *Matwons *and *Animatrices *and who supervised the community-based nutritional rehabilitation program;

• Mobile clinics at which an *Auxiliare *(auxiliary nurse) visited isolated communities every 1–2 months to provide basic curative and family planning services and to refer patients when indicated; and,

• 7 fixed primary health care facilities (Dispensaries and Health Centers), where curative care, immunizations and family planning services were provided.

Prenatal care and family planning services were free and available at Mobile Clinics, Health Centers, and in the Hospital Outpatient Clinic.

HAS also was operating a comprehensive program of tuberculosis detection and treatment, with over 400 patients entering treatment annually and only 7% failing to complete their entire course of treatment. The initial two months of treatment were provided at a residential facility in Deschapelles, and the follow-up treatment was provided at home through a directly observed treatment-short course (DOTS) program carried out by 12 *Accompagnateurs *who were working throughout the UCS District.

The HAS Hospital had 190 beds at that time and an active outpatient clinic, providing specialty referral care in adult medicine, pediatrics, obstetrics-gynecology, and surgery for patients within the UCS District and beyond. The health care system facilitated referral of patients to a higher point of care within the district and counter-referral down to lower levels as appropriate. Care was decentralized as much as possible and was designed with the goal of providing Rally Posts within a 30-minute walk of all households and Mobile Clinics with a one-hour walk. Even so, much of the population, particularly that segment living in the mountains, was more than three hours from a Dispensary or Health Center and even further away from the HAS Hospital.

At the time of the study, HAS was serving a population that was similar to the rest of Haiti in socio-economic terms. The coverage of key child survival services was considerably higher for the HAS service population than for the rest of Haiti, and under-five mortality had been at least half that compared to the rest of Haiti for the three previous decades [32,33]. The leadership of the institution and its staff were aware of the geographic and socioeconomic disadvantages which burdened the people living in the mountains, were concerned about the possibilities of health and health care inequities and assisted in the design of the study.

This article describes the findings arising from a comparison between the peripheral mountainous areas and the more central plains regarding access to, quality of, and coverage of health services and also regarding levels of childhood malnutrition and mortality within a well-developed and relatively high-quality health system as it existed during the period 2000–2003. We define health inequities here as "differences [in health] which are unnecessary and avoidable but, in addition, are also considered unfair and unjust [34].

## Methods

In 1999 and 2000, the Board of Directors at HAS mandated a comprehensive evaluation of all programs at HAS. The data used in this analysis arose from five different studies that HAS and collaborating organizations carried out as part of that evaluation (see also Table 1).

**Table 1:** Summary of Data Collection Methods Used for the Current Study

**Year**	**Type of Survey**	**Survey Area**	**Sample Size**	**Selection Criteria**
1999–2000	Retrospective pregnancy history including ages at death of live births	Households in the Primary Health Care Service Area of HAS	3,427 Women	10% sample of all reproductive age women in the Primary Health Care Service Area
2000	Household knowledge, practices and coverage survey	Households in the Primary Health Care Service Area of HAS and in the remainder of the UCS District	• 1,310 household heads • 1,295 women of reproductive age • 945 mothers/guardians of at least one child 0–59 months of age • 1,363 men 15–54 years of age	Sample reflective of the size of the respective populations in each of the Functional Units, with oversampling of the mountainous areas
2000	Quality of health services survey: • Patient exit interviews • Household interviews • Observations of patient encounters in the health facilities • Health staff interviews • Focus group discussions with Health Agents	Hospital and Primary Health Care Service Area of HAS	108 exit interviews 190 household interviews 6 focus group discussions with 42 health agents	• Exit interviews with caretakers following a child health visit at a health facility • Exit interviews with women visiting a health facility for prenatal care or family planning • Households and Health Agents were not randomly selected
2000	Assessment of hospital admission rates by Functional Unit (by analyzing data from the health information at HAS)	HAS hospital records for all admissions during the year 1999 were reviewed	1,210 hospital records	10% random sample
2003	Assessment of factors facilitating or obstructing the use of prenatal care (by analyzing data from the health information at HAS)	HAS Functional Unit	240 women who were pregnant in the year 2002	• 50 women were selected randomly from the mountainous areas • 50 women were selected randomly from the plains

(1) In 1999 and 2000, HAS staff carried out a household survey of 3,427 women of reproductive age in the HAS Primary Health Care Service Area and obtained a history from them regarding all births and subsequent mortality of live-born children. These women represented a 10 percent sample of the women of reproductive age. The pregnancy history survey identified 149 deaths of children less than five years of age during the previous five years.

(2) In the year 2000, a Haitian health research organization (the Institute of Child Health) conducted a household survey of the UCS District concerning a broad set of health and development issues. This survey included 1,310 household heads, 1,295 women of reproductive age, 945 mothers/guardians of at least one child 0–59 months of age, and 1,363 men 15–54 years of age. The households were selected in such a way that ensured adequate representation of both the mountainous and non-mountainous areas of the UCS District as well as of both the HAS Primary Health Care Service Area and the other seven Functional Units in the District. Here, we report findings from the HAS Primary Health Care Service Area only.

(3) In 2000, a study was carried out regarding the quality of services provided by the Community Health Division at HAS. This assessment involved exit interviews with caretakers at the time of 108 visits of children to health facilities and with 98 women patients at time of visits for prenatal care or family planning. Observations of encounters in the health facilities were carried out and health staff members at these facilities were interviewed. Also included were 190 household interviews and three focus group discussions with a total of 42 Health Agents. These households and Health Agents were not randomly selected.

(4) In 2003, a study was conducted to determine factors affecting the use of prenatal care[35]. In this study, data were obtained from the Community Health Division's health information system for one of HAS's Functional Units, which has 21,638 residents, about one-third of whom live in mountainous areas and the rest in the central valley plains. In this study, 240 women were recorded as being pregnant in 2002. Out of this number, 50 women were randomly selected from within mountainous communities and another 50 from the plains. Each community in this Functional Unit was characterized according to the following criteria: estimated walking time to the Health Center and whether or not Mobile Clinics come to the community.

(5) The HAS information systems were used to calculate the per capita utilization of inpatient hospital services by Functional Unit.

Mortality rates were calculated by dividing the number of deaths in the appropriate age group by the number of live births during the same time period and multiplying by 1,000. Exact Fisher 95 percent confidence intervals were calculated according to the methods developed by Fleiss [36] (1981) using Computer Programs for Epidemiologists (PEPI) version 4.0 [37].

## Results

### Inequities in Access to, Quality of, and Coverage of Health Services

#### Community Health Services

Among a convenience sample of 190 households in three Functional Units, a marked difference was observed in the frequency of home visits by Health Agents depending on whether they were working in the plains or in the mountains. In the Liancourt Functional Unit (in the plains), only 11 percent of the households reported that their Health Agent made fewer than five visits during the previous 12 months, while in the predominantly mountainous areas of Bastien and Plassac, 37 percent of households reported fewer than five visits.

In one Functional Unit (Plassac) in which both mountainous and non-mountainous communities were present, only 38% of the communities in the mountainous areas had a Mobile Clinic coming to the community compared to 98% of the non-mountainous communities, even though the walking distance to the Health Center was at least three hours for 60% of those living in mountainous communities while none of the communities in the plains were that far away. However, none of the pregnant women living in the plains in Plassac used a Mobile Clinic for their source of prenatal care compared to 18% of the pregnant women living in the mountainous communities. Living at least three hours walking time from the Health Center was significantly associated with lack of access to prenatal care (with lack of access defined as obtaining no prenatal visits), both in a bivariate as well as in a multivariate analysis [35].

The assessment of the quality of care provided at Dispensaries and Health Centers showed deficiencies, and these were more pronounced in the mountainous Functional Units. Family planning was not a frequent topic with patients at the time of prenatal visits. Counseling for breastfeeding as part of the prenatal visit was also infrequent. Overall, fewer than half of the women interviewed who had obtained prenatal care were given advice regarding breastfeeding.

Health Agents had not been trained to target men along with women in helping couples to make a joint and informed choice of family planning methods. HAS was not able to offer a broad choice of readily available family planning methods in the mountainous areas since intra-uterine devices and tubal ligation were available only in the Hospital.

Growth cards for monitoring the nutritional status of children were often incorrectly filled out. Childhood diarrhea was one of the major reasons for health facility utilization and, according to focus group discussions with Health Agents, diarrhea was the leading cause of childhood illness. Although mothers had been taught how to prepare and use oral rehydration fluid at home, more serious cases required treatment at a health facility. However, there was no provision for mothers to begin immediately rehydrating their child with diarrhea upon arrival at a Dispensary or Health Center, and the waiting time was as long as five hours.

Mothers in mountainous areas frequently reported that they were not feeding colostrum to their newborns, that they were weaning their children before two years of age, and that they were not feeding their children frequently. Hand washing with soap was often not practiced.

Immunization coverage of both children and women of reproductive age was significantly lower in the mountains than in the plains as was the contraceptive prevalence rate (Table 2)

**Table 2:** Comparisons of Coverage of Health Services in the Mountains and Plains of the Hôpital Albert Schweitzer Primary Health Care Service Area, 1999–2000

**Indicator**	**Mountains**	**Plains**	**Level of statistical significance of difference in the indicator in the mountains and plains**
Childhood immunization rate (percentage of children 12–23 months of age who had obtained all recommended immunizations)	72.9% (43/59) [59.7–83.6]	92.3% (48/52) [81.5–97.9]	p < 0.05
Contraceptive prevalence rate (percentage of women of reproductive age reporting use of a modern method of contraception)	21.5% (67/311) [17.1–26.5]	32.6% (313/959) [29.7–35.7]	p < 0.05
Hospital admissions (number of admissions per 1,000 population in 1999)	3.2 per 1,000 [2.8–3.6]	6.5 per 1,000 [5.9–7.0]	P < 0.01
Admission to the Hospital's malnutrition ward (number of admissions per 1,000 population in 1999)	1.41 (118/83,428) [1.20–1.70]	0.44 (33/74,999) [0.30–0.60]	p < 0.01

95% confidence intervals are shown in brackets.

#### Hospital Services

The rate of admission of patients to the HAS Hospital according to the Functional Unit in which the patient was living varied substantially according to the distance from the Hospital. The Functional Units closest to the Hospital (Deschapelles, Liancourt and Plassac) had admission rates of 6.4 admissions per 1,000 population per year, compared to rates of less than half that in the other four Functional Units (Table 2). The Gabriel Functional Unit, located in the most distant mountainous area, had the lowest utilization rate, with 2.5 admissions per 1,000 population. The rate of admission to the Hospital's malnutrition ward, on the other hand, was more than three times as great for children from the mountainous areas compared to those living in the plains (Table 2).

### Inequities in Health Status

#### Malnutrition in Children

The prevalence of moderate or severe malnutrition (as determined by the percentage of children with a weight for age and a height for age more than two standard deviations below the mean of a normal population) was more than twice as great in the mountainous areas as in the plains (Table 3).

**Table 3:** Comparisons of Health Status in the Mountains and Plains of the Hôpital Albert Schweitzer Primary Health Care Service Area

**Indicator**	**Mountains**	**Plains**	**Level of statistical significance of difference in the indicator between the mountains and plains**
Prevalence of malnutrition (children 6–59 months of age)			
Weight/age > 2 SD below median of standard	32.6% (79/242) [26.8–38.9]	12.6% (33/262) [8.8–17.2]	p < 0.01
Height/age > 2 SD below median of standard	50.4% (122/242) [43.9–56.9]	19.8% (52/262) [15.2–25.2]	p < 0.01
Number of births, 1996–2000	805	1,585	Not applicable
Total number of deaths among children 0–59 months of age, 1996–2000	73	79	Not applicable
0–4-year mortality rate, 1996–2000*	90.7 (73/805*1,000) [71.8–112.7]	49.8 (79/1,585*1,000) [39.7–61.7]	p < 0.01

*Expressed as deaths per 1,000 live births

95% confidence intervals are shown in brackets.

#### Under-five Mortality

The overall mortality among children aged less than five years in populations of the predominantly mountainous Functional Units was almost twice that for those in the plains: the 0–4-year mortality rate was 90.7 per 1,000 live births in the mountains and 49.8 in the plains (Table 3).

#### Fertility

The total fertility rate was 1.7 times greater in the Functional Units located in the mountainous areas than in the Functional Units located in the plains (6.8 versus 4.1).

### Socio-economic Differences

The mountainous population is socio-economically different from the population living in the valley area. While many statistics could be cited to characterize this difference, the different level of literacy is perhaps as notable as any. In the mountains, the level of illiteracy is twice as great as in the plains (70.9% versus 38.3%). The percentage of homes in the mountains with no electricity, with no latrine or indoor sanitation, and with no access to a protected source of water is also notably higher than among the homes in the plains (Table 4).

**Table 4:** Socio-economic Differences Between the Mountains and Plains of the Hôpital Albert Schweitzer Primary Health Care Service Area

**Indicator**	**Mountains**	**Plains**	**Level of statistical significance of difference between mountains and plains for the indicator**
Percentage of population 15 years and older with no formal education	70.9 (1,332/1,878) [68.8–73.0]	38.3 (697/1,820) [36.1–40.6]	p < 0.01
Percentage of homes with no electricity	99.9 (695/696) [99.2–100.0]	79.3 (487/614) [75.9–82.5]	p < 0.01
Percentage of homes with no latrine or indoor sanitation	87.1 (606/696) [84.3–89.5]	45.8 (281/614) [41.8–49.8]	p < 0.01
Percentage of homes with no access to a protected source of drinking water	95.6 (665/696) [93.7–97.0]	63.4 (389/614) [59.4–67.2]	p < 0.01

95% confidence intervals are shown in brackets.

Source: 2000 household knowledge, practices and coverage survey

## Discussion

There are probably few health and development programs in the world that have given more long-term emphasis on reaching the entire service population with basic services than HAS. Nonetheless, in spite of HAS's long-standing commitment to equity, marked socio-geographic differences in program inputs, access to services, utilization of services, coverage of services, and health status persist within the same relatively small service area (approximately 18 miles by 18 miles) administered by a single health program.

Over the past four decades, HAS has made it a priority to provide a similar level of intensity of primary health care services in the mountainous areas as in the valley plains. However, these data indicate that similar levels of inputs have not been successful in achieving health equity within the HAS service area. This is because the health needs are greater in the mountainous areas, and the inputs required to achieve the same health outcomes in the mountainous areas are much greater not only because of the greater health needs but because the difficulties of program operations are much greater (as a result of the difficult terrain, the greater dispersion of the population, the lower level of education and socio-economic status of the population, and the higher levels of childhood malnutrition).

The monitoring and evaluation system had not been designed previously to detect these disparities. While the leadership and staff of HAS noted that the results of the study affirmed their earlier concerns about possible health inequities between those living in the mountains and those living in the plains and continued their efforts to adapt programs to emerging needs.

There were a similar number of Health Agents (who make routine home visits) per population throughout the HAS service area even though in the mountainous areas, the health needs were greater, the distances between houses were greater, and the climbing of steep hills and mountains were required in order to reach all homes. All households were supposed to be visited every two months. Since the same number of Health Agents per population existed in the isolated mountainous areas as in the plains, then it was not surprising that there were fewer visits per household per year in the mountainous areas because of the considerable longer distances between houses and the steep climbs that were required while carrying equipment and materials for weighing children and dispensing vaccines.

In theory, the provision of Mobile Clinics was supposed to be concentrated in the most distant areas where the travel time to a Dispensary or Health Center was two or more hours. In practice though, this was unfortunately not the case.

The HAS Hospital has had a long-standing policy not to turn away patients with life-threatening illnesses because of inability to pay. Elective surgical procedures, however, required pre-payment, although the fees were among the lowest charged in Haiti. The Hospital did not operate a transportation system or ambulance service, and patients were expected to provide their own transport to the Hospital. Obviously, the need for hospital services was just as great (if not greater) in the mountainous areas as in the plains, but per capita hospital utilization by those living in the mountains was in fact only half that by those living in the plains.

One commonly cited reason among patients in the mountains for not accepting referral for hospitalization was that they said they cannot afford even the cost of transportation to get there (Ferdinand M. Evaluation of the Hôpital Albert Schweitzer Dispensaries. Unpublished document, 1998). Presumably, with their lower educational levels, mountain people did not possess the same level of understanding about what the HAS Hospital could do to assist them when certain types of illnesses and conditions arose. Or, perhaps they did not want to risk dying in a distant, unfamiliar place away from family and friends. Here is an opportunity for increasing the flow of information to the people in the mountains.

### More Recent Efforts at HAS to Reduce Disparities

Obvious initial steps for reducing the disparity in access to basic services included providing more training and support to the *Animatrices *in the mountainous areas and increasing the number of Health Agents in the mountainous areas. Previously, while each *Animatrice *had been assigned to 15 households, there had not been a close monitoring of their work by the Health Agents. For many years until the *Animatrice *program emerged, the Health Agents had been the prime home visitors, at which time they collected relevant data to be forwarded to the information system. But it became clear that they were unable to fulfill those expectations, to a large extent because of the extreme difficulties of coping with the rough mountainous terrain and geographical dispersion of the families.

Following the equity review in 2003, HAS changed the roles and interactive relationships in such a way that the *Animatrices *became the primary home visitors and the Health Agents gave more attention to supervising the work of the *Animatrices*. The *Animatrices *were distinctly interested in taking on these additional responsibilities and were proud to be considered for such roles.

A key new responsibility was to be the reporting by the *Animatrices *of their findings and actions in relation to their home visiting. The capabilities of the *Animatrices *for reporting, despite their high rates of illiteracy, were to be enhanced by both training and providing them with reporting charts that included artistic illustrations of various health conditions – diarrhea, respiratory infections, use of family planning, injuries, and more – that the *Animatrices *could complete by making a simple "check" mark. Thus, every household could be visited on a monthly or bimonthly basis with reporting to Health Agents through this simple report form. This home visiting and reporting process, including its supervision, was to be included in the information system. Further, it was envisioned that periodic group discussions among the *Animatrices *and Health Agents, including the community at times, would add to the oversight capacities of the system.

A further concern emerging from this equity review was the high rate of illiteracy and malnutrition in the mountains, which were seen as principle underlying causative factors for the higher child mortality there. A major nutrition initiative, which also included literacy training for mothers, began soon after this equity review, and this initiative gave priority to people living in mountainous areas. Future assessments will be required to determine to how effective these new activities have been in reducing health disparities.

These data provide compelling evidence that, from the standpoint of equity ("to each according to his or her need"), more intense services to mothers and children in the mountainous areas are required, with a particular focus on reducing malnutrition and improving access to hospital care for sick children when needed. The contribution of higher fertility to higher infant and child mortality has been well documented [38]. The higher fertility in the mountains no doubt contributes (along with increased rates of malnutrition) to the higher rates of infant and child mortality observed there. Therefore, the findings point to the obvious need to intensify family planning efforts in the mountainous regions as well.

HAS has recently undertaken major revisions in its organizational, financial and service components, in large part due to reductions in funding available to the institution. Even though the resources required to fully address the inequities identified in this study remain insufficient, there is nonetheless a clear and continuing commitment of the institution's leadership to the pursuit of equity and to the equity-related concepts and processes described in this paper.

### The Rationale for Addressing Health Inequities at the Local Level

There are at least three rationales for addressing issues of health inequities at the local level. First, ethics demands it. Distributive justice requires special concern for those who are worst off or most disadvantaged [39].

Secondly, if a health program is serious about improving the health of the population it serves, it becomes obvious that the best way to achieve this is by putting more resources into those segments of the population where the prevalence of disease and death are the highest, since improvements in health in this segment of the population will lead to greater improvements in the health of the entire population than would an investment which is equally spread across the entire population.

Thirdly, the magnitude of the health gap is often a major rather than a minor one. In many developing countries around the world, differences in under-five mortality between rich and poor households are unacceptably high and, in some areas, are becoming wider [40]. Mothers and children in poorer households are exposed to greater health risks than those with better socio-economic circumstances, and poorer children often have less resistance to disease because of greater levels of malnutrition. These inequities are compounded by reduced access to preventive and curative health care services.

Experience and evidence about how to reduce inequities among the poor segments of populations are growing [41]. Successful approaches include those that improve geographic access to health interventions in poor communities, those that subsidize health care and health inputs for the poor, and those that empower poor communities.

Regular monitoring of inequities and using the resulting information for education, advocacy, and increased accountability among the poorest and most marginalized persons in the service area are urgently needed. Equity must be a priority in the design of health delivery strategies, and mechanisms must exist to ensure accountability at local, national and international levels for addressing and reducing inequities.

The levels of inequities observed at HAS are substantial, and they are within a single small geographic area served by a single, relatively well-developed health program committed to equity for more than 50 years. Thus, these findings suggest that health inequities might be even more substantial in geographic areas in poor countries where there are less well-developed health systems and where the same commitment to equity does not exist. Monitoring of equity and reducing inequity should become priorities for local health programs [42].

## Conclusion

We have demonstrated marked health inequities within a local population which had ready access to a well-developed health care program with relatively high utilization, high coverage of services, substantial childhood mortality impact, and a long-term commitment to equity. Even within such a relatively well-designed and well-administered comprehensive health system, a monitoring system is needed to detect inequities in access to and coverage of health services, not to mention inequities in health status. Given that health is a basic human right, equity in health should have "pride of place" [43]. Health programs need to develop ongoing monitoring and evaluation systems that enable inequities to be uncovered. Leaders of health programs need to ensure that corrective actions are undertaken to continue efforts at reducing health inequities. Special attention needs to be given to monitoring and evaluation in more remote areas where the needs are likely to be greater, where geographic and socio-economic barriers are more pronounced, and where health personnel are often less well-trained and less well-supported. Local political and governmental support for these initiatives will be needed. Thus, equity assessment and actions taken to promote equity based on such an assessment can, in the end, contribute to equitable outcomes in health and, thereby, enhanced human well-being.

## Abbreviations

HAS Hôpital Albert Schweitzer

UCS *Unite Communale de Sante *(Communal Health unit, or rural health district)

## Competing interests

The author(s) declare that they have no competing interests.

## Authors' contributions

HP conceived of the idea for this paper, assisted in the collection of some of the data, participated in some of the data analysis, and contributed to the writing of the paper.

LK-S performed a literature review of equity issues, collected and analyzed the data pertaining to prenatal care utilization, and helped to draft the manuscript. AA collected and analyzed some of the data cited here and helped to draft the manuscript. JB provided guidance in the conceptual framework used here, provided encouragement at all stages of this project, and helped to draft the manuscript. All authors read and approved the final manuscript.
